# Copper-transporting ATPase is important for malaria parasite fertility

**DOI:** 10.1111/mmi.12461

**Published:** 2013-12-12

**Authors:** Sanketha Kenthirapalan, Andrew P Waters, Kai Matuschewski, Taco W A Kooij

**Affiliations:** 1 Max Planck Institute for Infection Biology, Parasitology Unit10117, Berlin, Germany; 2 Wellcome Trust Centre for Molecular Parasitology, Glasgow Biomedical Research Centre, University of GlasgowGlasgow, G12 8TA, Scotland, UK; 3 Institute of Biology, Humboldt University10117, Berlin, Germany

## Abstract

Homeostasis of the trace element copper is essential to all eukaryotic life. Copper serves as a cofactor in metalloenzymes and catalyses electron transfer reactions as well as the generation of potentially toxic reactive oxygen species. Here, we describe the functional characterization of an evolutionarily highly conserved, predicted copper-transporting P-type ATPase (CuTP) in the murine malaria model parasite *Plasmodium berghei*. Live imaging of a parasite line expressing a fluorescently tagged CuTP demonstrated that CuTP is predominantly located in vesicular bodies of the parasite. A *P. berghei* loss-of-function mutant line was readily obtained and showed no apparent defect in *in vivo* blood stage growth. Parasite transmission through the mosquito vector was severely affected, but not entirely abolished. We show that male and female gametocytes are abundant in *cutp*^−^ parasites, but activation of male microgametes and exflagellation were strongly impaired. This specific defect could be mimicked by addition of the copper chelator neocuproine to wild-type gametocytes. A cross-fertilization assay demonstrated that female fertility was also severely abrogated. In conclusion, we provide experimental genetic and pharmacological evidence that a healthy copper homeostasis is critical to malaria parasite fertility of both genders of gametocyte and, hence, to transmission to the mosquito vector.

## Introduction

Copper is a trace element essential to all eukaryotic life and functions as a cofactor in many key enzymes, such as mitochondrial cytochrome *c* oxidase and superoxide dismutase. Its properties as a transition metal enable the electron transfer by metalloenzymes and yet make it toxic to cells (Festa and Thiele, 2011; Hodgkinson and Petris, 2012; Samanovic *et al*., 2012). Consequently, the redox property of copper can catalyse the production of hydroxyl radicals under aerobic conditions through the Fenton reaction leading to oxidative damage to proteins, DNA, lipids, etc. (Jomova and Valko, 2011). Therefore, copper homeostasis needs to be tightly controlled and cells express a range of copper-specific transporting and sequestering proteins (Lutsenko, 2010; Argüello *et al*., 2012).

*Plasmodium* species are unicellular ancient eukaryotic parasites that are the sole cause of malaria. These obligate intracellular parasites harbour at least three genes, which are predicted to be involved in copper transport and the maintenance of a healthy copper homeostasis: two proteins harbouring a putative Ctr copper transporter domain (Pfam04145; PBANKA_102150 and PBANKA_130290) (Martin *et al*., 2009; Choveaux *et al*., 2012) and one copper-transporting P-type ATPase (CuTP; PBANKA_041650) (Rasoloson *et al*., 2004). The sources of copper for parasites during blood stage development are unknown. The relatively abundant red blood cell derived copper superoxide dismutase taken up along with haemoglobin in the parasite food vacuole was suggested as one possible source (Rasoloson *et al*., 2004). Alternatively, one or both of the putative Ctr copper transport proteins could be involved in import. Localization of the *P. falciparum* copper transport protein (PF3D7_1439000) to the erythrocyte and parasite plasma membranes could support a role in either import or export (Choveaux *et al*., 2012).

The effect of intracellular depletion of Cu^+^ can be tested with the cell-permeable copper chelator neocuproine (2,9-dimethyl-1,10-phenanthroline; CID 65237) (Smith and McCurdy, 1952), which is highly specific for Cu^+^. In a previous study, addition of neocuproine to cultured blood stage *Plasmodium falciparum*, the parasite responsible for the majority of malaria-related deaths, was reported to inhibit ring-to-trophozoite transition but did not affect infectivity of schizonts (Rasoloson *et al*., 2004). The identity of copper containing enzymes and their importance for blood stage development remains unknown, particularly since *Plasmodium* parasites lack an orthologue of Cu/Zn superoxide dismutase.

The *P. falciparum* CuTP was localized to both parasite and host cell membrane and it was suggested that it mediates Cu^+^ efflux in order to minimize toxic effects of excess Cu^+^ (Rasoloson *et al*., 2004). There are two CuTP homologues in human and mouse genomes, *ATP7A* and *B*, and mutations therein are linked to Menkes and Wilson disease respectively (La Fontaine *et al*., 2010). Both transporters usually reside at the trans-Golgi network where they facilitate the biosynthesis of cuproenzymes. In addition, they can detoxify by exporting excess intracellular Cu^+^ (La Fontaine and Mercer, 2007; Hasan and Lutsenko, 2012).

Here, we have used the murine malaria model *Plasmodium berghei* to investigate the *in vivo* role(s) of this evolutionarily highly conserved transport protein during the malaria parasite life cycle. We demonstrate that *PbCuTP* is dispensable for blood stage development and important only for male and female fertility. Localization of the fluorescently tagged protein to vesicular bodies suggests that such bodies might serve as copper storage organelles throughout the *Plasmodium* life cycle.

## Results

### Apicomplexan parasites encode evolutionary conserved CuTP proteins

We identified genes encoding CuTP in all queried *Plasmodium* species and some related apicomplexan parasites, e.g. *Cryptosporidium parvum* and *Toxoplasma gondii* (Table S1). All proteins contained at least one predicted metal-ion scavenging motif (MxCxxC) at the amino-terminal end and at least three pairs of predicted transmembrane domains. In addition, the intramembranous ‘CPC’ and ‘MxxSS’ motifs required for the selective translocation of Cu^+^ ions across membranes (Argüello, 2003) are conserved in all apicomplexan CuTP sequences placing them into the family of intracellular P_1B1_-type ATPases (Fig. S1). Phylogenetic analysis of selected CuTP protein sequences from a variety of model organisms and species with a biological or evolutionary link to malaria parasites confirmed an ancestry reflecting the known evolutionary relationships (Fig. S2). Together, consistent presence of CuTP in all parasitic and free-living eukaryotes indicates important functions.

### CuTP is expressed in all *Plasmodium* life cycle stages and localizes to vesicle-like structures

**Figure 1:**
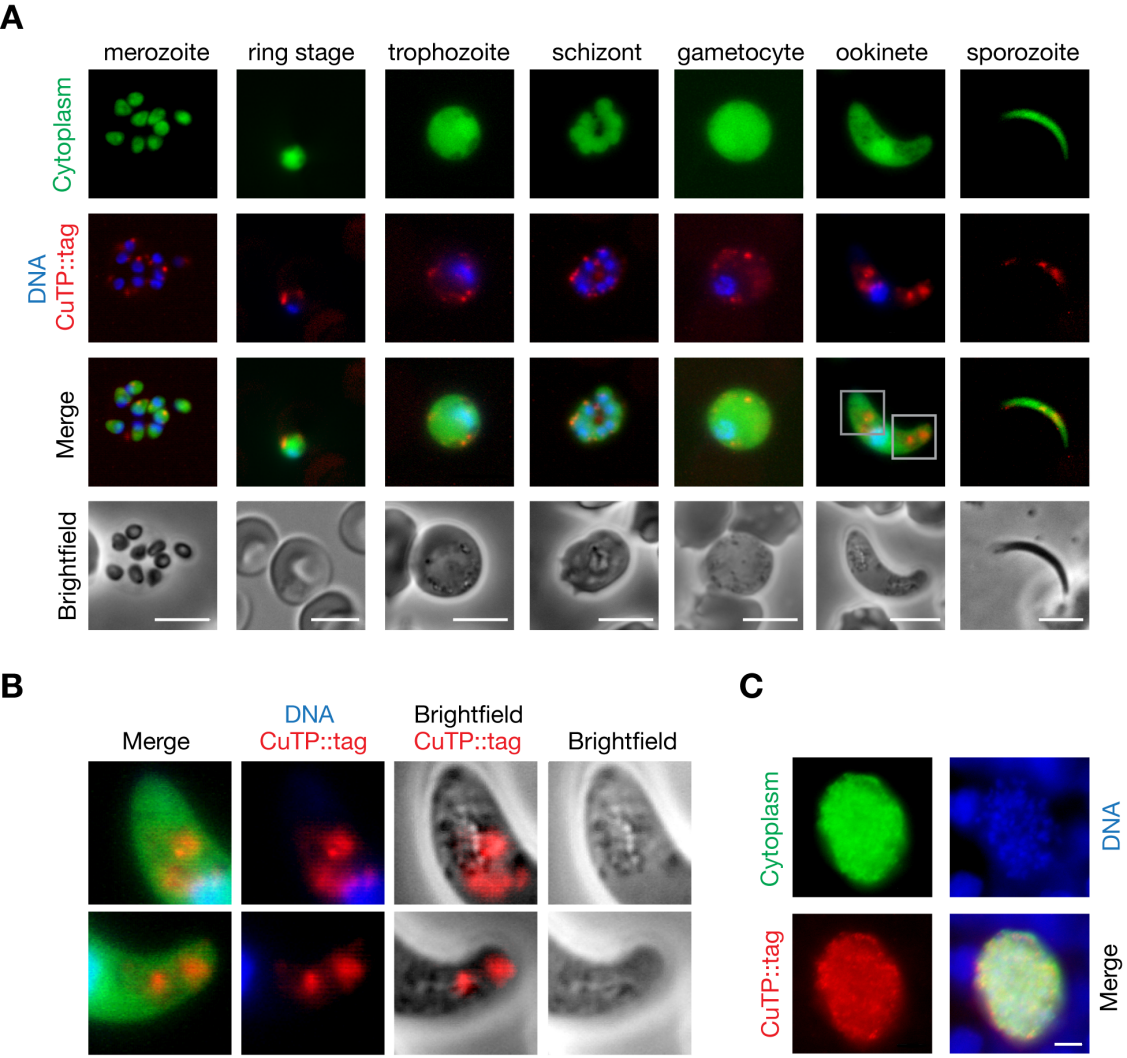
CuTP is expressed in all parasite stages and localizes to vesicle-like structures inside the parasite.
Live fluorescent imaging of *cutp::tag* parasites reveals abundant CuTP expression in all parasite stages examined, including intracellular asexual and sexual blood stages, and extracellular ookinetes and salivary gland sporozoites. Note that CuTP consistently localizes to vesicle-like structures inside the parasite. Bars, 5 μm.Higher magnification of CuTP-positive vesicle-like structures in ookinetes. The regions boxed in (A) exemplify the characteristic localization of the CuTP-mCherry signal to one or several large vesicles we observed in all analysed life cycle stages.Immunofluorescence of fixed *cutp::tag* liver stage parasites with an mCherry antibody. Shown is an infected hepatoma cell 48 h after infection. Bar, 5 μm.

To examine the temporal and spatial expression of CuTP, we generated a transgenic parasite line where the endogenous CuTP was carboxy-terminally fused to a red fluorescent mCherry-triple c-myc hybrid protein (Fig. S3A). This was achieved by tailored double cross-over homologous recombination, generating stable recombinant parasites. Transfection of WT parasites, followed by flow-cytometric isolation of recombinant parasites yielded an isogenic parasite line, termed *cutp::tag* (Fig. S3B). Live imaging of *cutp::tag* parasites revealed abundant expression of *Pb*CuTP in asexual and sexual blood stages, ookinetes, and sporozoites (Fig. 1A). Intriguingly, in all intra-and extracellular parasite stages the tagged CuTP consistently localized to large vesicle-like structures inside the parasite cytoplasm (Fig. 1B). This was most striking in ookinetes, where CuTP localizes to multiple vesicle-like structures of unknown origin. In addition, staining with an anti-mCherry antibody on fixed liver stage parasites 48 h after infection revealed intracellular expression of CuTP similar to the other parasite life cycle stages (Fig. 1C). Western blot analysis demonstrated integrity of the fusion protein and excluded potentially aberrant localization of a processed carboxy-terminal fluorescent tag, at least in mixed blood stages (Fig. S3C). We conclude that CuTP is present throughout the entire *Plasmodium* life cycle and localizes to intraparasitic structures that might represent storage vesicles.

### Localization of *Toxoplasma gondii* CuTP to intraparasitic structures

We initially hypothesized that CuTP might localize to acidocalcisomes, acidic organelles rich in calcium and phosphorus that can be found in *Plasmodium* species as well as *T. gondii* (Miranda *et al*., 2008; Moreno and Docampo, 2009). Since an antibody against the *T. gondii* plant-like vacuolar proton pyrophosphatase (VP1) that localizes to acidocalcisomes and a plant-like vacuole (Miranda *et al*., 2010) was not cross-reactive with *P. berghei* blood stage parasites (data not shown), we generated a stable recombinant *T. gondii* line expressing endogenous CuTP (TGGT1_020170) fused in-frame with a 2xMyc tag using single cross-over homologous recombination (Fig. S4).

**Figure 2:**
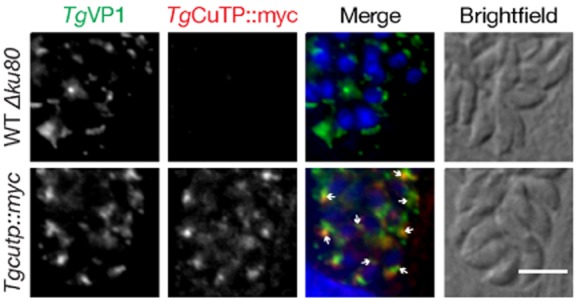
*Tg*CuTP localizes in close proximity of the acidocalcisomes and/or plant-like vacuole in *Toxoplasma gondii*. Shown are immunofluorescence micrographs of fixed intracellular WT Δ*ku80* and *Tgcutp::myc* tachyzoites labelled with antibodies against c-Myc and the plant-like vacuolar proton pyrophosphatase (*Tg*VP1), a marker for acidocalcisomes and the plant-like vacuole. Arrows indicate areas where *Tg*CuTP and *Tg*VP1 are localized in close juxtaposition. Hoechst 33342 was used to stain DNA (blue in merge). Bar, 5 μm.

Double immunofluorescence of intracellular *Tgcutp::myc* parasites with the anti-*Tg*VP1 and anti-Myc antibodies revealed that *Tg*CuTP localizes to intraparasitic structures juxtaposed to the acidocalcisomes and/or plant-like vacuole (Fig. 2). This spatial distribution closely reflects our findings in *P. berghei*, indicative of potential functional similarities of the CuTPs in the two apicomplexan parasites. Although we could not provide definitive assignment of the parasite organelle, we did not detect CuTPs at the parasite plasma membranes, in good agreement with their phylogenetic placement as intracellular P_1B1_-type ATPases.

### Targeted deletion of *CuTP* does not affect asexual blood stage growth

**Figure 3:**
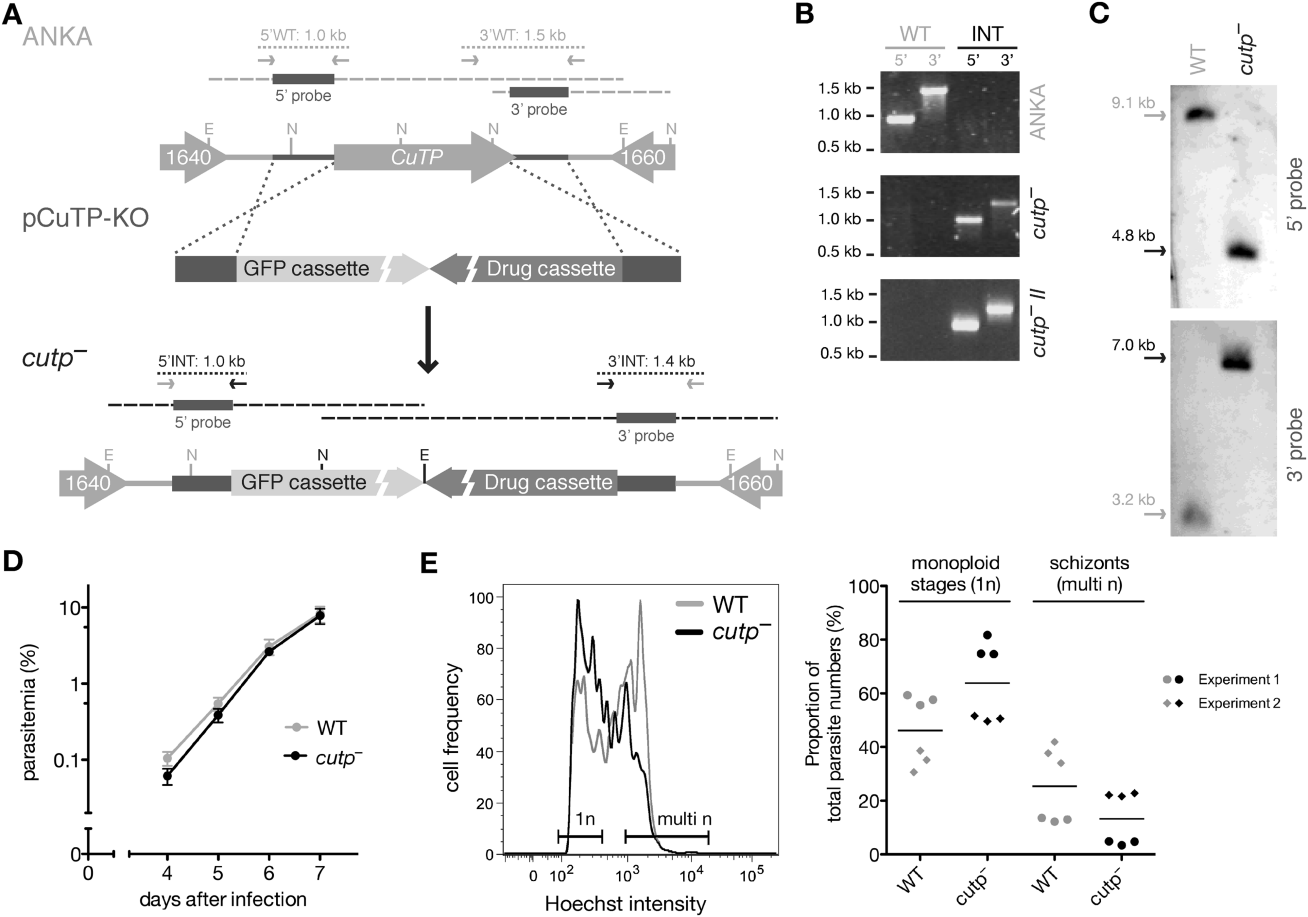
Population distribution in *cutp*^−^ blood stage parasites is affected but not *in vivo* growth.
Schematic representation of the generation of *cutp*^−^ parasites. *CuTP* is located on chromosome 4 between PBANKA_041640 and PBANKA_041660 (ANKA). The targeting vector (pCuTP-KO) and the predicted recombinant locus (*cutp*^−^) are also indicated. Using a double cross-over homologous recombination strategy, *CuTP* was replaced with highly expressing GFP-and drug-selectable cassettes. Primer pairs for diagnostic PCRs and expected fragment sizes are indicated by arrows and dotted lines respectively.Diagnostic PCRs to demonstrate successful integration (INT) of pCuTP-KO in two independent transfection experiments and absence of WT contamination following flow cytometric sorting of two isogenic *cutp*^−^ lines (*cutp*^−^ and *cutp*^−^ *II*).Southern blot analysis of the *cutp*^−^ line used for most experiments. The 5′ and 3′ probes as indicated in panel (A) were used to hybridize EcoRV and NdeI restriction-digested gDNA, respectively, revealing the expected size shifts.Blood stage growth of *cutp*^−^ (black) and WT (grey) parasites. NMRI mice (*n* = 3) were intravenously injected with 1000 infected erythrocytes and parasitemia monitored by microscopic examination of Giemsa-stained thin blood smears. WT and *cutp*^−^ parasites blood stage development did not differ significantly over the time-course or at individual time points (*P* > 0.05; two-way ANOVA followed by Bonferroni post-tests).*In vitro* maturation of asexual blood stages appears affected in *cutp*^−^ parasites. Displayed is a representative flow cytometry plot that illustrates the distribution of DNA content in cultured asexual blood stages (left). An accumulation of monoploid (ring and trophozoite, 1n) stages, and, as a consequence, fewer multiploid (schizont, ≥ 8n) stages can be observed in the *cutp*^−^ (black) as compared with WT (grey) parasites. Quantification of monoploid and schizont stages of two independent parasite cultures (circles or diamonds) with three replicates each (right).

We next wanted to study the *in vivo* role(s) of *CuTP* and generated a targeting vector, termed pCuTP-KO, to ablate *CuTP* in *P. berghei* (Fig. 3A). Successful generation of *cutp::tag* parasites already indicated that the gene locus is amenable to genetic manipulation. Employing a similar gene targeting strategy, we performed two independent transfections followed by flow-cytometric isolation of recombinant parasites. To our surprise, we readily obtained *cutp*^−^ parasite lines in both cases (Fig. 3A–C), indicating that loss of *CuTP* is compatible with asexual blood stage growth.

In order to directly compare *in vivo* blood stage growth, we infected mice (*n* = 3) by intravenous injection of 1000 infected erythrocytes of either *cutp*^−^ or WT parasites and monitored parasitemia side-by-side over the course of one week (Fig. 3D). We observed no difference in the exponential growth phase, and both *Plasmodium* lines resulted in similar high parasite burden.

We next synchronized *P. berghei* asexual parasites by high dose intravenous injection of culture-enriched schizonts. Blood was collected 2 h later for a highly synchronized *in vitro* parasite culture. Flow-cytometric analysis of WT and *cutp*^−^ parasites cultured for 20 h suggested a reduction of multi-nucleated schizonts in *cutp*^−^ parasites when compared with WT parasites in two independent experiments (Fig. 3E). However, this mild arrest did apparently not affect exponential propagation of asexual blood stages *in vivo* (Fig. 3D). Together, successful generation of two independent *cutp*^−^ parasite lines establishes a dispensable role of the copper-transporting ATPase during asexual blood infection.

### Additive inhibition by the cell-permeable copper chelator neocuproine

The observed reduction of *in vitro*-cultured schizonts in *cutp*^−^ parasites encouraged us to test whether depletion of intracellular copper could, at least partially, phenocopy genetic ablation of *CuTP*. We tested the effect of a broad range of neocuproine concentrations on schizont formation in an *in vitro* culture assay (Fig. S5). Though schizont formation in *cutp*^−^ parasites was typically reduced to 35–65% of WT at any given concentration, WT and *cutp*^−^ parasites were affected to a similar extent by increasing neocuproine concentrations. Negligible schizont formation was reached in WT and *cutp*^−^ parasites at 500 nM neocuproine.

### Severe impairment of natural transmission in *cutp*^−^ parasites

**Figure 4:**
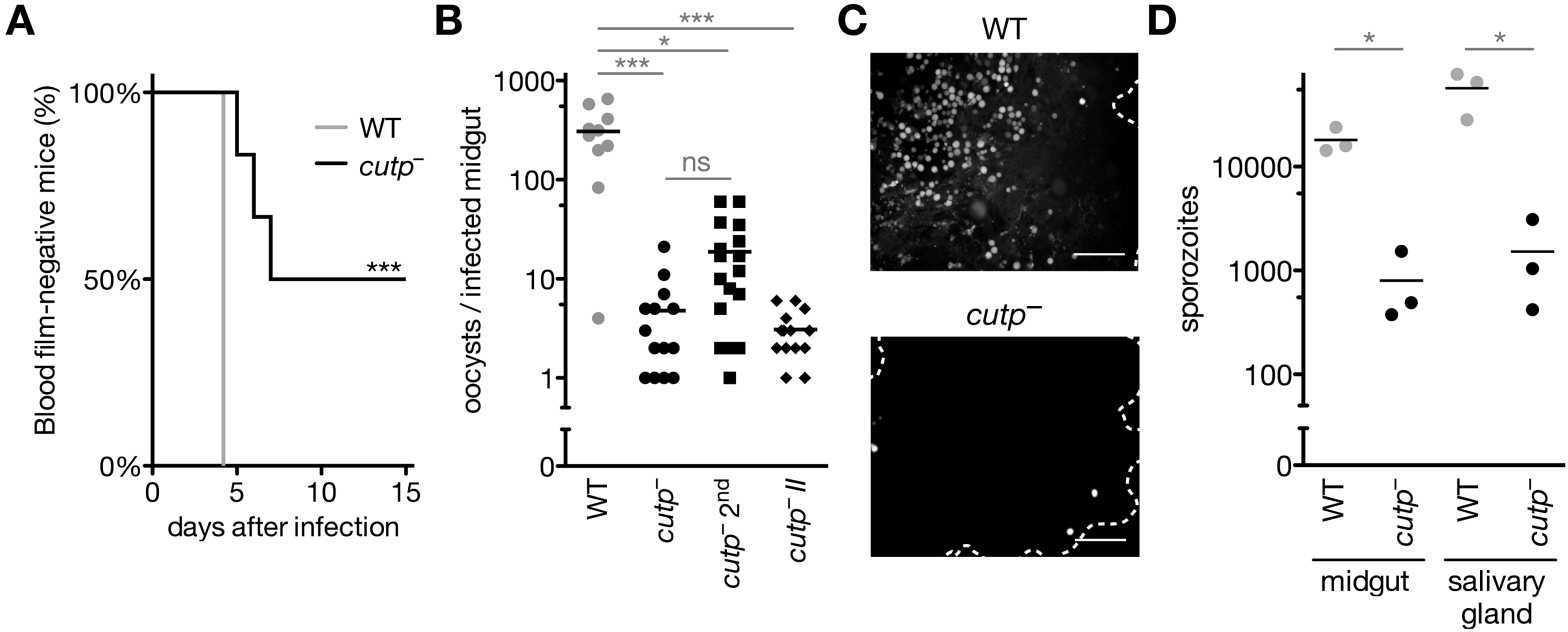
Defect of *cutp*^−^ parasites in natural *Plasmodium* transmission.
Kaplan–Meier analysis of mice exposed to WT-or *cutp*^−^-infected *Anopheles* mosquitoes. Mice (*n* = 6) were exposed to 10–12 female *Anopheles stephensi* mosquitoes, and parasitemia was followed by microscopic examination of daily Giemsa-stained thin blood films. Survival curves of WT and *cutp*^−^ differed significantly (***P < 0.001 for both Mantel–Cox and Gehan–Breslow–Wilcoxon tests).Quantification of oocysts per infected mosquito in two isogenic *cutp*^−^ parasite lines (*cutp*^−^ and *cutp*^−^ *II*) and WT parasites. Note that low oocyst numbers persist in a subsequent infection experiment following completion of the life cycle (2nd) (*P < 0.05; ***P < 0.001; ns, non-significant; Kruskal–Wallis test followed by Dunn's post-test).Representative images of mosquito midguts infected with WT (top) and *cutp*^−^ (bottom).Quantification of midgut-and salivary gland-associated sporozoites shows that sporozoite numbers in the *cutp*^−^ line are reduced compared with WT (*P < 0.05; non-parametric, one-tailed Mann–Whitney's test).

Since *CuTP* is dispensable for asexual blood stages, we next explored whether it plays an important role during life cycle progression. To this end, we first performed a natural transmission experiment (Fig. 4A). *Anopheles stephensi* mosquitoes were infected by feeding on *cutp*^−^-and WT-infected mice. On day 17, when sporozoites have colonized salivary glands, these mosquitoes were used to infect naïve mice. Monitoring of blood stage parasitemia revealed a severe defect in *cutp*^−^*-*infected mice (Fig. 4A). Mice infected with *cutp*^−^ sporozoites stayed either parasite-free or became blood-film positive with a 2-day delay on average, indicating a severe defect during *Plasmodium* life cycle progression.

We next dissected infected *Anopheles* mosquitoes and determined the oocyst burden (Fig. 4B and C). While WT-infected mosquitoes contained large numbers of oocysts, *cutp*^−^-infected mosquitoes displayed very few oocysts. Quantification of oocyst numbers in repeated feeding experiments with the two isogenic *cutp*^−^ parasite lines confirmed this finding (Fig. 4B). In order to test whether this phenotype is stable, we subjected *cutp*^−^ parasites selected by the first transmission experiment to a second transmission cycle. Again, oocyst numbers were dramatically reduced, indicating that loss of *CuTP* function results in a robust phenotype that cannot be swiftly adapted to. As expected, quantification of sporozoites in infected mosquitoes revealed low numbers (Fig. 4D), in good agreement with the severe defect in the transmission experiment. We conclude that the main defect in *cutp*^−^ parasites is efficient colonization of the insect vector, the definitive host of the *Plasmodium* life cycle.

### Male exflagellation is significantly reduced in *cutp*^−^ parasites

For the effective colonization of the anopheline mosquito midgut and subsequent successful transmission to a new vertebrate host, malaria parasites need to go through a round of sexual reproduction (Kooij and Matuschewski, 2007). This complex process involves the activation of dormant gametocytes in the mosquito midgut. Upon emergence from the red blood cell highly motile male microgametes aim to fertilize a female macrogamete.

**Figure 5:**
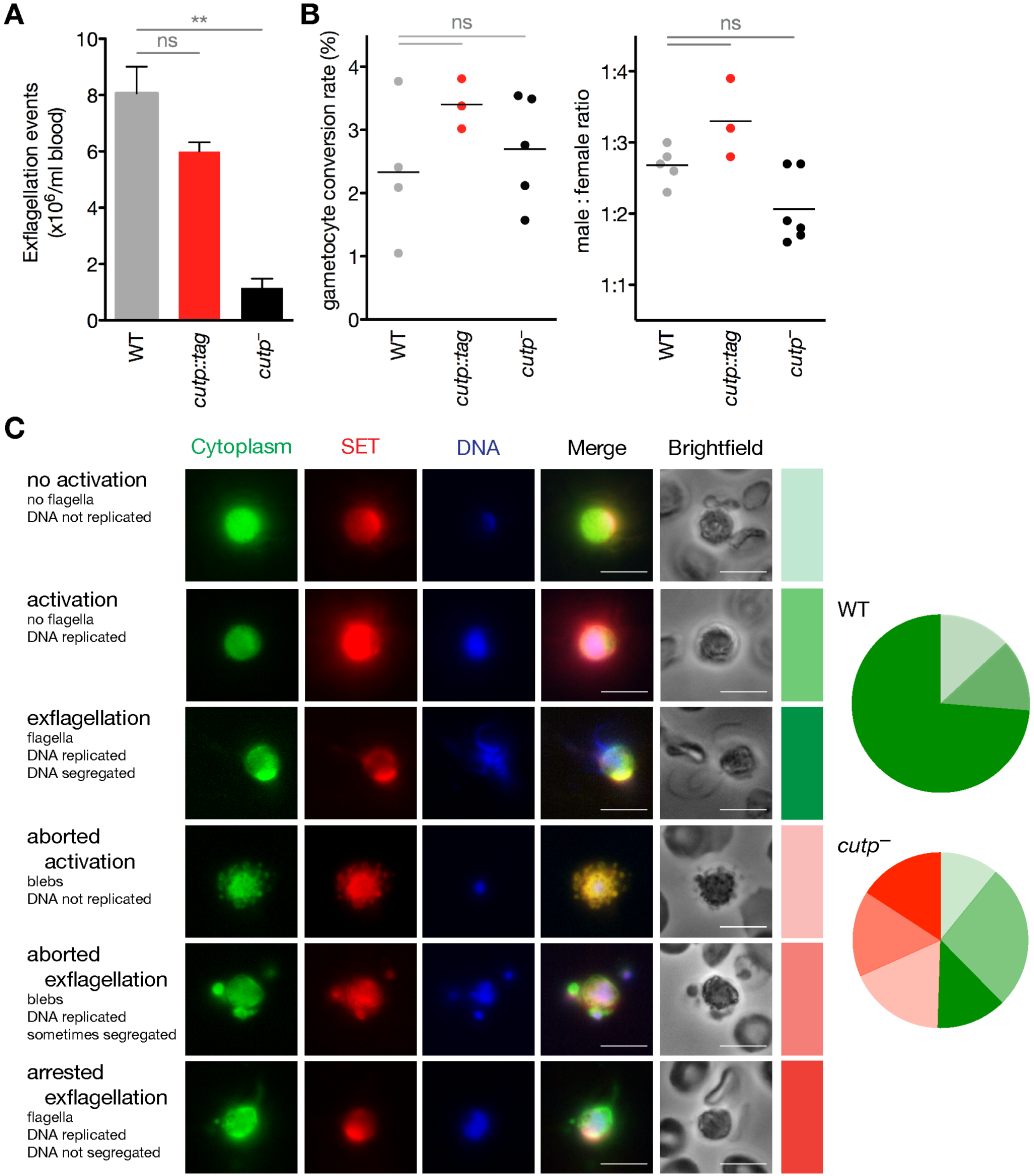
Male microgamete formation is defective in *cutp*^−^ parasites.
Quantification of exflagellation events *in vitro* (**P < 0.01; ns, non-significant; Kruskal–Wallis test followed by Dunn's post-test).*In vivo* gametocyte conversion rate (left) and ratio between female and male gametocytes (right) did not differ significantly (ns, non-significant; Kruskal–Wallis test followed by Dunn's post-test).Immunofluorescence analysis of activated male gametes. Parasites were fixed 14 min after activation and stained with anti-heat shock protein 70 antibody (cytoplasm). Male gametes were identified by staining with anti-SET antibody and Hoechst for DNA. The presence or absence of DNA replication, DNA segregation, formation of flagella, and the formation of blebs were used to monitor the health status of the male gametes and to define six categories as described to the left of the exemplary immunofluorescence images. Proportions of healthy (shades of green) and abnormal (shades of red) gametocytes were scored for WT (*n* = 30) and *cutp*^−^ (*n* = 56) parasites according to these categories that are represent in the pie charts by the colour coding as it appears to the right of the images. Bars, 5 μm.

To establish whether a fertility defect might be the cause of the low *cutp*^−^ oocyst numbers, we quantified the number of exflagellating male gametocytes (Fig. 5A). Exflagellation activities of both *cutp*^−^ lines were reduced to 10–15% of the activity of WT parasites. In contrast, *cutp::tag* male gametocytes showed exflagellation levels within the WT range (Fig. 5A). This finding demonstrated that the fluorescently tagged CuTP is functional and the observed targeting to intracellular vesicles (Fig. 1A and B) is physiologically relevant.

Of note, no differences between WT and *cutp*^−^ lines in either gametocyte production or ratios of male and female gametocytes (Fig. 5B) were detected. Immunofluorescent microscopic analysis of activated male *cutp*^−^ gametes revealed the presence of healthy and degenerated males (Fig. 5C). Together, our findings establish that the transmission defect in the absence of *CuTP* can be, at least partially, attributed to impaired microgamete activation and/or formation.

### Inhibition of Cu^+^ impairs exflagellation of wild-type gametes

**Figure 6:**
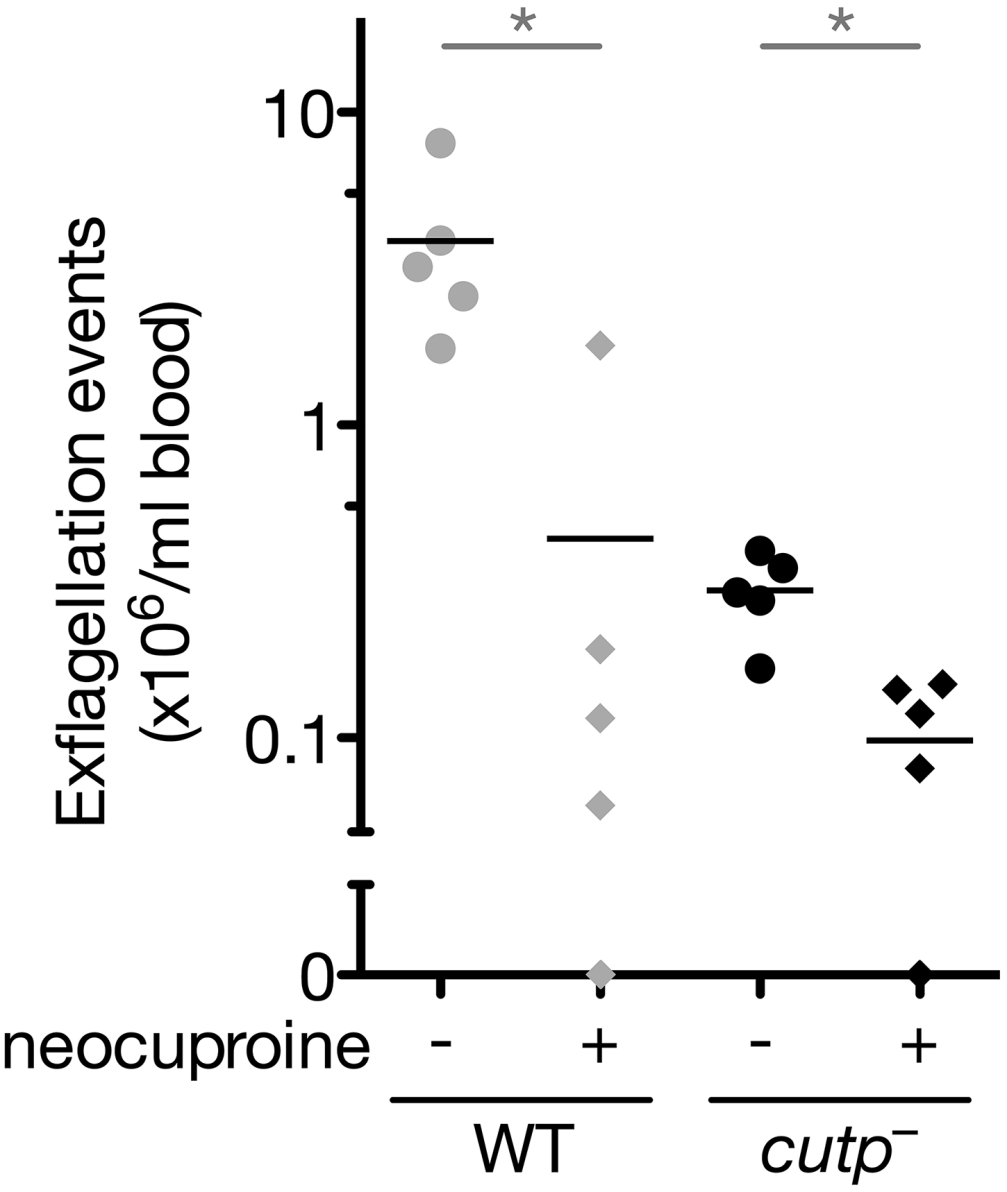
Reduction of male microgamete exflagellation in the presence of the cell-permeable Cu^+^-chelator neocuproine.Shown are the numbers of exflagellating WT and *cutp*^−^ male gametocytes in the presence or absence of 1 μM neocuproine (*P < 0.05; non-parametric, two-tailed Mann–Whitney's test).

To further substantiate the link between male fertility and copper, we investigated the effects of copper ion addition and chelation on exflagellation in an *in vitro* assay. Chelation of intracellular Cu^+^ with neocuproine in WT parasites resulted in a substantial reduction of male exflagellation, mimicking the defect observed in *cutp*^−^ parasites (Fig. 6). In contrast, we were not able to restore the exflagellation deficit of the *cutp*^−^ parasites or neocuproine-treated WT parasites by addition of extracellular Cu^+^ or Cu^2+^ (data not shown).

### Ablation of *CuTP* impairs female fertility

**Figure 7:**
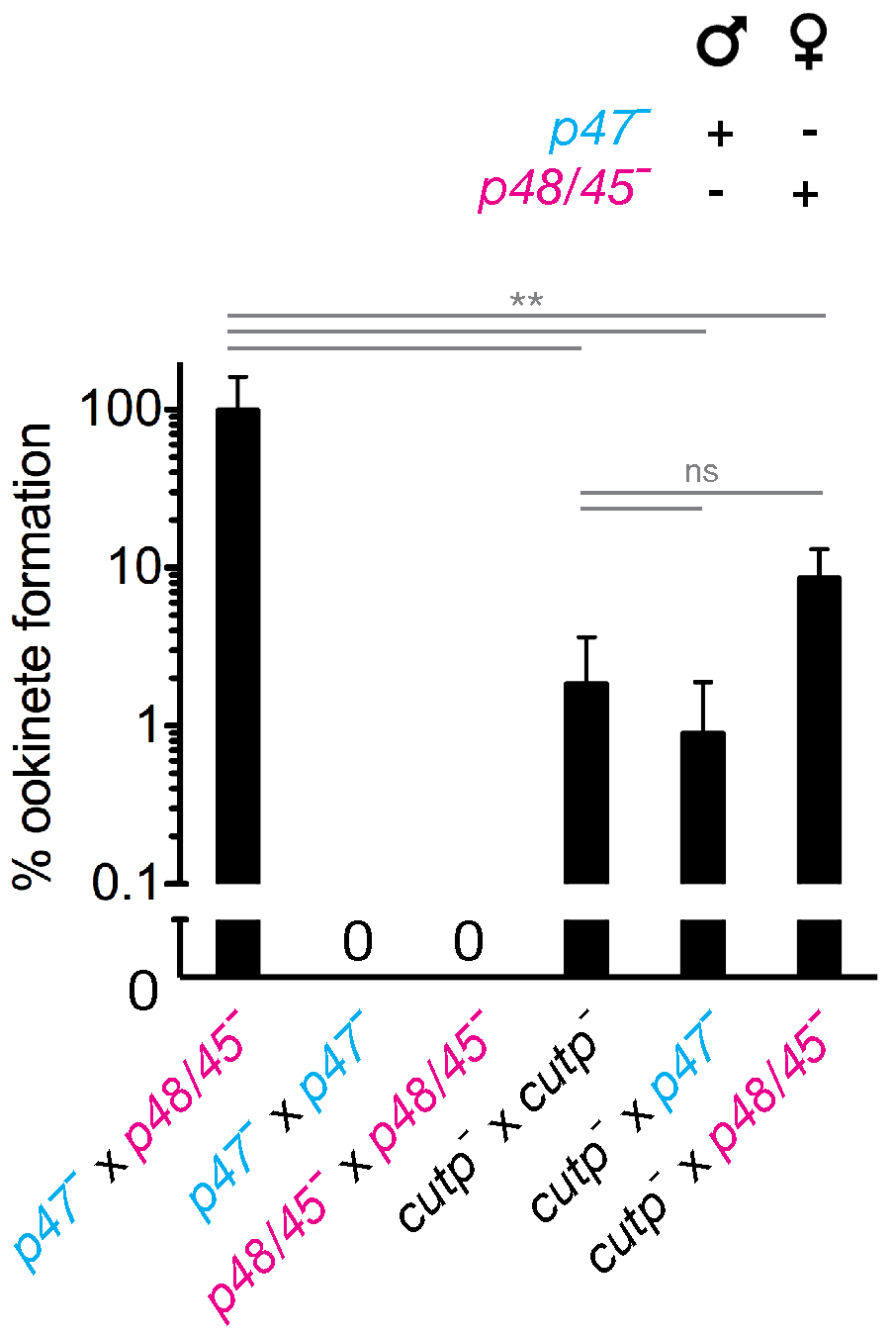
*Cutp*^−^ parasites display reduced female fertility. Cross-fertilization assay to determine fertility of female and male *cutp*^−^ gametes. The fertilization partners are *p48/45*^−^ (pink), which produces only healthy female gametes and *p47*^−^ (blue), which produces only healthy males. As positive and negative controls, a *p47*^−^ × *p48/45*^−^ cross-fertilization and homologous ookinete cultures were included. Ookinete cultures of *cutp*^−^ parasites (black) yielded only 2% of the reference culture. A similar low level of ookinetes resulting from cross-fertilization with healthy male gametes (*p47*^−^) suggests a defect in female fertility of *cutp*^−^ parasites. Partial rescue in cross-fertilization with healthy females (*p48/45*^−^) corroborates male gamete deficiency of *cutp*^−^ parasites. Results were obtained from three independent experiments (repeated measures analysis of variance with Bonferroni's post-test; **P < 0.01; ns, non-significant).

So far, we determined a ∼ 10-fold reduction in microgamete formation, yet observed a ∼ 100-fold reduced oocyst burden in *cutp*^−^ parasites. To confirm a male fertility defect and test female fertility, we performed a cross-fertilization assay and quantified the resulting ookinetes, a functional read-out for gamete fertility (van Dijk *et al*., 2001; 2010,). As published previously, no ookinetes were detectable in cultures containing *p48/45*^−^ or *p47*^−^ parasites alone, whereas in co-cultures *p47*^−^ male microgametes were able to productively fertilize *p48/45*^−^ females and ookinetes were readily formed (Fig. 7). Next, we tested ookinete formation in cultures containing *cutp*^−^ parasites alone. Ookinetes were observed only infrequently, approximating 2% of WT ookinete numbers (Fig. 7). This prominent reduction correlates with the very low oocyst formation (Fig. 4B), and apparently exceeds reduced exflagellation rates (Fig. 5A).

Strikingly, co-culturing *cutp*^−^ parasites with fertile *p47*^−^ males did not result in higher ookinete numbers (Fig. 7). A likely interpretation of this result is that healthy male gametes in *p47*^−^ were unable to compensate for the reduced exflagellation activity of *cutp*^−^ parasites, indicative of an additional female fertility defect. Indeed, co-cultures of *cutp*^−^ and fertile *p48/45*^−^ female parasites yielded approximately 10% ookinetes (Fig. 7), strongly indicating a partial rescue of female fertility defects in *cutp*^−^ females by addition of *p48/45*^−^ parasites. Together, our cross-fertilization studies establish that *Plasmodium* CuTP is critical only for fertility of male and female gametes.

## Discussion

### *Plasmodium berghei CuTP* functions in parasite fertility

In this study, we establish an important function for the putative *Plasmodium* Cu^+^-transporting P_1B1_-type ATPase in parasite fertility. Thus far, very little is known about possible links between fertility and copper homeostasis in any eukaryotic organism. A first indication for a potential correlation between copper levels and spermatozoal motility was noted in a study that compared highly and less motile human sperm cells (Battersby and Chandler, 1977). More recently, a redundant copper transporter was shown to play an important role in male fertility in *Drosophila melanogaster* (Steiger *et al*., 2010). In male mice, *Atp7a* mutants displayed abnormal testes morphology and decreased male gamete quantity and quality, including motility and membrane integrity (Kowal *et al*., 2010). Together with our findings, a universal link between copper homeostasis and male fertility emerges.

Thus far, copper transporters have not yet been implicated in female fertility. Malaria parasites lack sex-specific chromosomes. Instead, sex-specific gene expression is regulated by transcriptional and translational control mechanisms (Kooij and Matuschewski, 2007). Our analysis of temporal and spatial CuTP expression revealed a remarkably uniform expression and localization to intracellular vesicles. Therefore, we consider regulation of *Plasmodium CuTP* expression of minor, if any, importance. However, loss of *CuTP* function results in specific defects in male and female fertility *in vitro* and, as a consequence, in severe impairment of colonization of the definitive invertebrate host *in vivo*. While this unexpected finding offers new opportunities for transmission blocking strategies, additional experimentation is warranted to elucidate the roles of Cu^+^ and, most likely, one or more critical cuproenzymes in gamete fertility. It is tempting to hypothesize that copper homeostasis might also play previously unrecognized roles in female fertility of other eukaryotes.

### Copper export or storage?

At present, it is unknown, whether *Plasmodium* CuTP mediates copper export or intracellular redistribution. A previous study proposed a role for *P. falciparum* CuTP in copper export and localization of the protein to the erythrocyte plasma membrane (Rasoloson *et al*., 2004). A second *P. falciparum* copper transport protein (PF3D7_1439000) thought to mediate copper transport has also been localized to the plasma membrane of ring-stage infected red blood cells (Choveaux *et al*., 2012). Accordingly, the observed phenotype of *CuTP* loss-of-function parasites might be explained by a defect in copper export, leading to toxic accumulation and excessive generation of reactive oxygen species. Our data demonstrate a predominant localization at rounded vesicular bodies inside the cytoplasm of all life cycle stages as well as an intraparasitic localization in *T. gondii*. This localization is more consistent with an important role in intracellular copper redistribution or storage, most likely for biosynthesis of cuproenzymes. We cannot entirely rule out residual *Pb*CuTP targeting to the parasite vacuolar and plasma membranes or the erythrocyte plasma membrane (Fig. 1). However, our data do not support such a localization. Normal exflagellation rates of the *cutp::tag* parasites suggests that the mCherry-3xMyc tagged protein is functional and is therefore expected to localize correctly. In fact, alternative interpretations of the single published immunofluorescence image, obtained with a polyclonal *Pf*CuP-ATPase antiserum, are possible. We note a strong vesicular-like pattern, reminiscent of the localization of the endogenously tagged protein, which was not addressed by the authors (Rasoloson *et al*., 2004). In conclusion, we favour a central role of CuTP in intracellular copper homeostasis and, possibly, export via vesicles, comparable to ATP7 in higher eukaryotes (La Fontaine and Mercer, 2007; Hasan and Lutsenko, 2012).

### What is the CuTP-labelled vesicle-like compartment?

In the absence of validated subcellular markers or antibodies to perform colocalization studies and assign a definitive identity to the *Pb*CuTP-labelled vesicle-like structures, we note that in *T. gondii* CuTP associates with the acidocalcisomes and/or the plant-like vacuole (Miranda *et al*., 2010). Copper has not yet been unequivocally reported in these organelles, in marked contrast to other essential transition metals, such as zinc and iron. Intriguingly, X-ray microanalysis of acidocalcisomes showed a significant copper peak, which was, however, attributed to the copper grids used (Scott *et al*., 1997; Ruiz *et al*., 2004). Nevertheless, presence of copper in acidocalcisomes could not be ruled out either.

Alternatively, the vesicles may be part of a trans-Golgi network where *Pb*CuTP could assist the biosynthesis of cuproenzymes, similar to mammalian ATP7A and B at low or basal copper concentrations (La Fontaine and Mercer, 2007; Hasan and Lutsenko, 2012). It is tempting to speculate, that some as yet unidentified cuproenzyme is critical to malaria parasite fertility thus leading to the observed phenotype. At high copper levels, ATP7A and B relocate and sequester excess copper in vesicles, which eventually fuse to the plasma membrane (Hasan and Lutsenko, 2012). In asexual blood stage, we observed that the CuTP-labelled vesicles were often at the parasite periphery, which could be an indication for a second function in copper detoxification, thus linking the observation that *P. falciparum*-infected erythrocytes contain lower copper levels than uninfected ones (Rasoloson *et al*., 2004) with the intraparasitic localization of CuTP.

Finally, the CuTP-labelled structures may represent novel as yet unidentified (storage) organelles. The distinct patterns of the CuTP-labelled vesicle-like structures in different phases of the *Plasmodium* life cycle ranging from a singular, small juxta-plasma membrane spot in ring stages to several large vesicles in ookinetes, together with a similar intraparasitic stain in the related parasite *T. gondii*, clearly merits further studies to resolve the cellular function(s) of this compartment.

Taken together, our data provide strong evidence for a link between copper homeostasis and malaria parasite fertility. Although malaria parasites have a highly specialized, obligate intracellular life style, our experimental genetics findings illustrate that a *Plasmodium* protein with the key signatures of a Cu^+^-transporting P_1B1_-type ATPase is particularly important for male and female fertility.

## Experimental procedures

### Experimental animals

This study was carried out in strict accordance with the German ‘Tierschutzgesetz in der Fassung vom 22. Juli 2009’ and the Directive 2010/63/EU of the European Parliament and Council ‘On the protection of animals used for scientific purposes’. The protocol was approved by the ethics committee of the Berlin state authority (‘Landesamt für Gesundheit und Soziales Berlin’, permit number G0469/09). C57BL/6 mice were used for sporozoite infections. All other parasite infections were conducted with NMRI mice.

### Generation and imaging of *Tgcutp::myc* parasites

For the generation of *Tgcutp::myc* parasites, we used single cross-over homologous recombination. Briefly, *T. gondii* RH Δ*ku80* parasites (Huynh and Carruthers, 2009) were cultivated on human foreskin fibroblasts (HFF) and free tachyzoites transfected with ∼ 30 μg linearized pTgCuTP-myc. Recombinant parasites were selected with 25 μg ml^−1^ mycophenolic acid and 40 μg ml^−1^ xanthine as described previously (Donald *et al*., 1996). Approximately one week later, HFF colonized coverslips were infected with either untransfected or transfected parasites.

For colocalization studies of *Tgcutp::myc* parasites, infected HFF cells were fixed two days after invasion with 4% paraformaldehyde in PBS, permeabilized with 0.2% Triton-X in PBS, blocked with 3% bovine serum albumin in PBS, and incubated with monoclonal mouse anti-myc antibodies (Santa Cruz Biotechnology, 1:1000 dilution) and rabbit anti plant-like vacuolar proton pyrophosphatase (VP1) antibodies (1:4000 dilution) (Miranda *et al*., 2010) in 3% BSA/PBS for 70 h at 4°C. Bound antibodies were detected using donkey anti-mouse Alexa Fluor 546 and goat anti-rabbit fluorescein conjugated antibodies respectively (1:1000 dilution; Invitrogen) in 3% BSA/PBS. DNA was visualized by Hoechst 33342 (Invitrogen).

### Generation of *cutp^−^* and *cutp::tag* parasites

We used the advanced gene replacement strategy (Janse *et al*., 2006; Kooij *et al*., 2012) to generate parasites with their *CuTP* deleted or tagged at the carboxy-terminus with a fluorescent protein-epitope tag. Details of the molecular cloning strategy to generate the two resulting plasmids, termed pCuTP-KO and pCuTP-tag, can be found in the *Supplemental Experimental Procedures* and Figs 2 and S3. The plasmids were verified by commercial Sanger sequencing. For transfection into wild-type *P. berghei* strain ANKA (WT) parasites, vectors were linearized with SalI and ScaI. Isogenic mutant parasite lines were isolated by flow cytometry 7–8 days after transfection (Kenthirapalan *et al*., 2012). Correct integration of the transfection vectors in the mutant parasite lines and absence of contaminating WT parasites was confirmed by diagnostic PCR (see *Supplemental Experimental Procedures* for details). The *cutp*^−^ line used for the majority of the experiments was analysed by Southern blot using the PCR DIG Probe Synthesis kit and the DIG Luminescent Detection kit (Roche), according to the manufacturer's protocol. The 5′ probe was amplified using primers CuTP-F2-SacII and CuTP-R1-EcoRI (see Table S2 for all primer sequences) and annealed to EcoRV restriction-digested gDNA, resulting in bands of 9.1 kb (WT) and 4.8 kb (*cutp*^−^). The 3′ probe was amplified using primers CuTP-F5-AvrII and CuTP-R4-KpnI and annealed to NdeI restriction-digested gDNA resulting in bands of 3.2 kb (WT) and 7.0 kb (*cutp*^−^).

Phenotypic analysis of the mutant parasite lines was done in direct comparison with two GFP-expressing parasite lines: Bergreen (Kooij *et al*., 2012) was used for all asexual and sexual blood stage development and ookinete cultures; GFPcon (Janse *et al*., 2006) was used for mosquito infections and liver stage development. As both lines display WT behaviour and life cycle progression, both are referred to as WT parasites.

### Analysis of *Plasmodium* life cycle progression

To compare blood stage development of *cutp*^−^ and WT parasites, 1000 infected erythrocytes were injected intravenously into naïve recipient NMRI mice. The progress of the infection was monitored by daily microscopic examination of Giemsa-stained thin blood smears. Gametocyte conversion rates, male:female gametocyte ratios, and exflagellation rates were determined at day 3 after intravenous infection of 10^7^ blood stage parasites into naïve NMRI mice. 5 μl tail blood was mixed with 125 μl RPMI (complemented with 50 μM xanthurenic acid), and an aliquot transferred into a Neubauer chamber for incubation at 20°C. Exflagellation centres were determined by microscopic observation at 400× magnification for 6 min, starting 12 min after incubation. For ookinete *in vitro* cultures, blood from infected mice at day 3 after infection with 10^7^ blood stage parasites was used. 1 ml infected blood obtained via cardiac puncture was immediately added to ookinete medium (RPMI 1640 with L-glutamine and 25 mM HEPES supplemented with 100 mM sodium bicarbonate, 125 U ml^−1^ penicillin/streptomycin, 10% fetal calf serum, and 50 μM xanthurenic acid, pH 8.0) and incubated for 16–20 h at 20°C. Ookinetes were purified with anti-P28 antibody-coated magnetic beads, and the number determined by microscopic observation (400×). *Anopheles stephensi* mosquitoes were raised at 28°C and 75% humidity under a 14 h light/10 h dark cycle. Mosquito stage development was analysed using standard techniques (Vanderberg, 1975). Briefly, to determine infectivity and the number of midgut sporozoites, mosquitoes were dissected on day 14 after feeding. The number of salivary gland sporozoites was determined on day 17. To determine the sporozoite infectivity to mice, naïve recipient C57BL/6 mice were infected by exposure to 10–12 infected mosquitoes. Patency was determined by microscopic examination of daily Giemsa-stained thin blood smears. Liver stages of *cutp::tag* parasites were cultured *in vitro* and analysed as described (Haussig *et al*., 2011). At 48 h after infection, cultured hepatoma cells were fixed and incubated with monoclonal mouse anti-*P. berghei* heat shock protein 70 (1:300 dilution) (Tsuji *et al*., 1994) and rat anti-mCherry (1:500 dilution; Chromotek) antibodies. Bound antibodies were detected using goat anti-mouse Alexa Fluor 488 and anti-rat IgG Alexa Fluor 546 conjugated antibodies respectively (1:2000 dilution; Invitrogen). Nuclei of live and fixed parasites were visualized with the DNA-dye Hoechst 33342 (Invitrogen).

### *Plasmodium berghei* exflagellation assay

*In vitro* exflagellation was analysed as described (Raabe *et al*., 2009). Briefly, mice were infected with 10^7^ WT or *cutp*^−^-infected blood stage parasites. Three days later mice were bled by heart puncture and aliquots of 50 μl infected blood were transferred immediately to 500 μl of preheated normal or supplemented gametocyte maintenance buffer (GMB: 4 mM NaHCO_3_, 20 mM glucose, 137 mM NaCl, 4 mM KCl, 1 mM MgCl_2_, 1 mM CaCl_2_, 20 mM HEPES, pH 7.24–7.29, 0.1% BSA). 1 μM neocuproine, 1 μM CuCl_2_ (for Cu^2+^), or 1 μM CuCl_2_ (incubated for 30 min with 1 mM ascorbic acid in order to obtain Cu^+^ ions) were tested. Following incubation at 37°C for 1 h, the infected red blood cells were collected by a short spin and resuspended in 1 ml RPMI (prewarmed at 20°C and complemented with 50 μM xanthurenic acid). 10 μl was immediately transferred to a Neubauer chamber and incubated at 20°C. Exflagellation centres were quantified by microscopic observation at 400× magnification from 12–18 min after activation.

### Image acquisition

All images were recorded on a Leica DMR or Zeiss AxioObserver Z1 epifluorescence microscope and processed minimally with ImageJ.
