# Supplementary material for: Copper-transporting ATPase is important for malaria parasite fertility
Source: Mol Microbiol. 2013 Dec 12;91(2):315–25. doi: 10.1111/mmi.12461 (PMC4016742; doi:10.1111/mmi.12461)
Supplement: Supplementary file 1 [file mmi0091-0315-sd1.pdf]

**Supplemental information for**

# Copper-transporting ATPase is important for malaria parasite fertility

Sanketha Kenthirapalan, Andrew P. Waters, Kai Matuschewski, and Taco W.A. Kooij

**Contents:**

- **Supplemental Figures S1 – S6**
- **Supplemental Table S1 and S2**
- **Supplemental Experimental Procedures**
- **Supplemental References**

## Kenthirapalan *et al.*, Supplemental Figure S1

```
                                 H6
            ------------------------
EcCopA      IATTVLIIACPCALGLATPMSIISGVGRAAEFGVLVRDADALQRASTLDTVVFDKTGT 527
ScCcc2p     TATSVVIVACPCALGLATPTAIMVGTGVGAQNGVLIKGGEVLEKFNSITTFVFDKTGT 631
AtPAA1      LSCSVLVVACPCALGLATPTAMLVGTSLGARRGLLLRGGDILEKFSLVDTVVFDKTGT 602
AtRAN1      FSISVVVIACPCALGLATPTAVMVATGVGATNGVLIKGGDALEKAHKVKYVIFDKTGT 662
HsATP7A     ASITVLCIACPCSLGLATPTAVMVGTGVGAQNGILIKGGEPLEMAHKVKVVVFDKTGT 1048
HsATP7B     TSITVLCIACPCSLGLATPTAVMVGTGVAAQNGILIKGGKPLEMAHKIKTVMFDKTGT 1031
LmCuTP      FFISTVVAACPCALGLATPTAIMVGTGVGAKNGVLVKSGTTLEEVRRVNCVVLDKTGT 801
CmCuTP      FALSVMAISCPCAIGITIPIVTLISTIKALNNDILVQSPHIFDSILKIKSVVFDKTGT 811
TgCuTP      FGIGVLSIACPCALGLAAPTALMVGTGVAARLGILVKSGQAFELATKLKALVLDKTGT 1236
PbCuTP      FSLSVLCVACPCAVGLASPLSIAISSYICSNIGIIIKNINIFEILLNCNHFIFDKTGT 1301
PfCuTP      FSLSILCVACPCAVGLASPLSIAISTYICSSIGIIIKNINIFEIFLECKHFIFDKTGT 1945
PvCuTP      FSLSILCVACPCAVGLASPLSIAISSYICSSIGIILKNINLFEIFLECNHFIFDKTGT 1431
                 :  :***::*:: *     .       .::::.   ::       .::*****

                                H7                         H8
            ----------------------        ----------------------
EcCopA      KQNLLGAFIYNSIGIPVAAGILWPFTGTLLNPVVAGAAMALSSITVVSNANR 826
ScCcc2p     KLNLFWALCYNIFMIPIAMGVLIPWG-ITLPPMLAGLAMAFSSVSVVLSSLM 949
AtPAA1      KQNLWWAFGYNIVGIPIAAGVLLPLTGTMLTPSMAGALMGVSSLGVMTNSLL 914
AtRAN1      RLNYVFAMAYNVVSIPIAAGVFFPVLRVQLPPWAAGACMALSSVSVVCSSLL 983
HsATP7A     RINFVFALIYNLVGIPIAAGVFMPIG-LVLQPWMGSAAMAASSVSVVLSSLF 1406
HsATP7B     RINLVLALIYNLVGIPIAAGVFMPIG-IVLQPWMGSAAMAASSVSVVLSSLQ 1372
LmCuTP      YGNFIWAFGYNLLMLPAASGLLYPFFHIRLPPVVAGAAMMLSSLSVLTSSLT 1148
CmCuTP      RSNLTWALVFNIVAIPLAAGILYP-R-VVIPPVAASLLMMLSSVIIILSSLS 1264
TgCuTP      RLSILWACIFNAAGIPLAAGAFYKFK-VFVPPTLAGAMMALSSVLVISNALM 1739
PbCuTP      KFNFLFSFFINIFFILLASGAFYAIN-FVFSPFHFTFLMFCSSIIVILSSLS 1942
PfCuTP      KFNFLFSFFINIFFILLASGAFYSLN-YVFSPFLFTFLMFCSSIIVILSSLS 2560
PvCuTP      KFNFLFSFLINVFFILLASGSFYALN-YVFSPFLFTFLMFCSSIIVILSSLS 2065
              .   :   *   :  * * :       . *      *  **: :: .:
```

**Fig. S1**. *Plasmodium* CuTP share motifs with $Cu^+$-specific $P_{1B1}$-type ATPases.

Alignment of the two regions containing the three most carboxy-terminal transmembrane domains (H6-8) of twelve representative $P_{1B1}$-type ATPases, which selectively transport monovalent copper or silver (Argüello, 2003). Highlighted with green is an aspartic acid residue conserved in all P-type ATPases. Conserved residues with potential metal-binding side chains are highlighted with cyan. The combined presence of the "CPC" and "MXXSS" motifs in H6 and H8, respectively, is a determinant of $Cu^+$-selectivity. Both motifs were found in all apicomplexan CuTP sequences. Table S1 lists all protein sequence IDs, the sources, species, and strains.

**Kenthirapalan *et al*., Supplemental Figure S2**

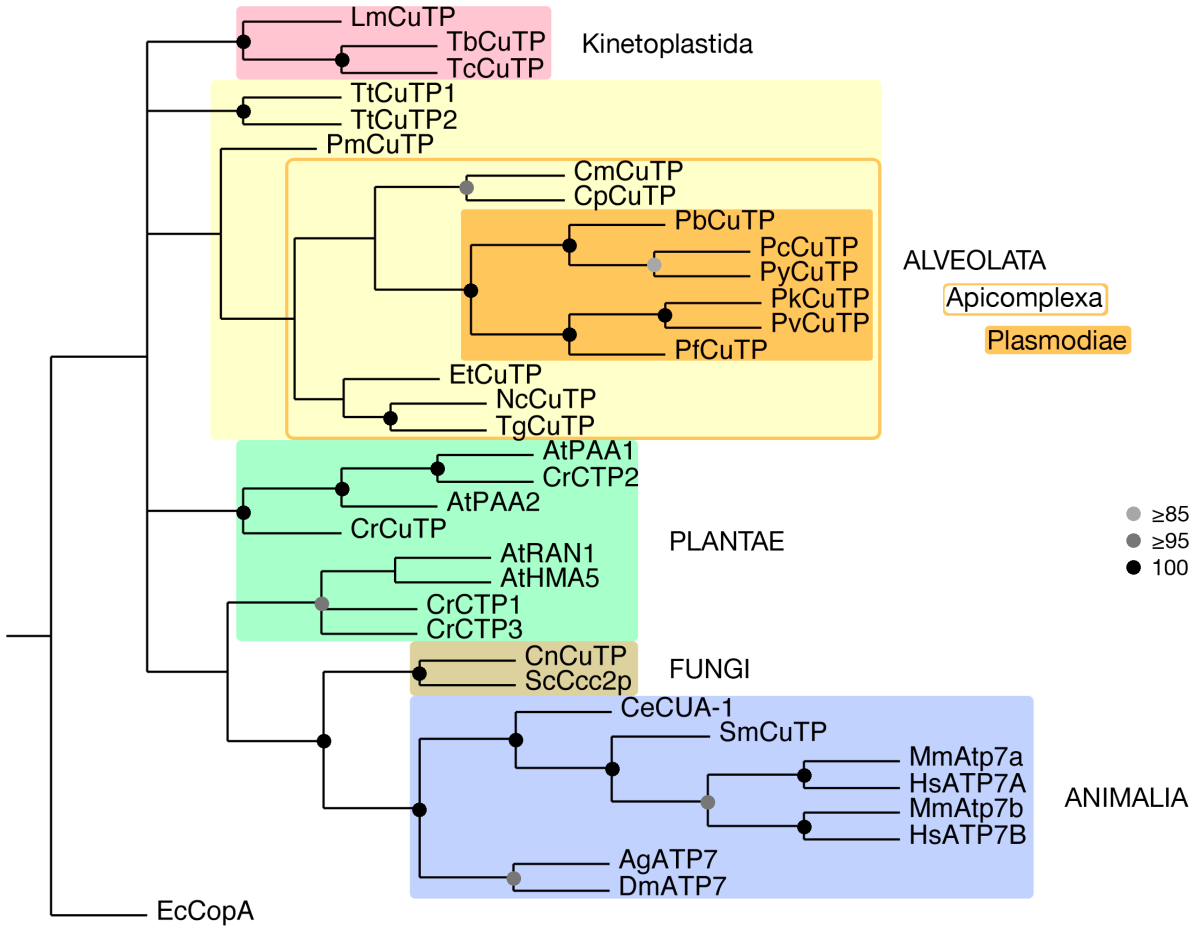


**Fig. S2**. Phylogenetic tree of CuTP proteins.

Most parsimonious phylogenetic tree of copper-transporting P-type ATPases from 26 eukaryotic organisms, including many common model and pathogenic species. The tree was built using *Escherichia coli* K12 CopA as the outgroup and nodes with bootstrap values <70 were collapsed. Dots indicate nodes with bootstrap values ≥85 (light gray), ≥95 (dark gray), or of 100 (black). All sequences clustered in kingdom specific clades, though both *plantae* and *alveolata* formed two distinct clades. All apicomplexan parasites formed a single clade with a highly confident subclade including all *Plasmodium* spp. Table S1 lists all protein sequence IDs, the sources, species, and strains. The sequences are grouped and shaded according to the major clades as they appear in the phylogenetic tree. Sequences from human pathogens are indicated in bold. The tree was constructed using the PHYLIP package (Felsenstein, 1996).

**Kenthirapalan *et al.*, Supplemental Figure S3**

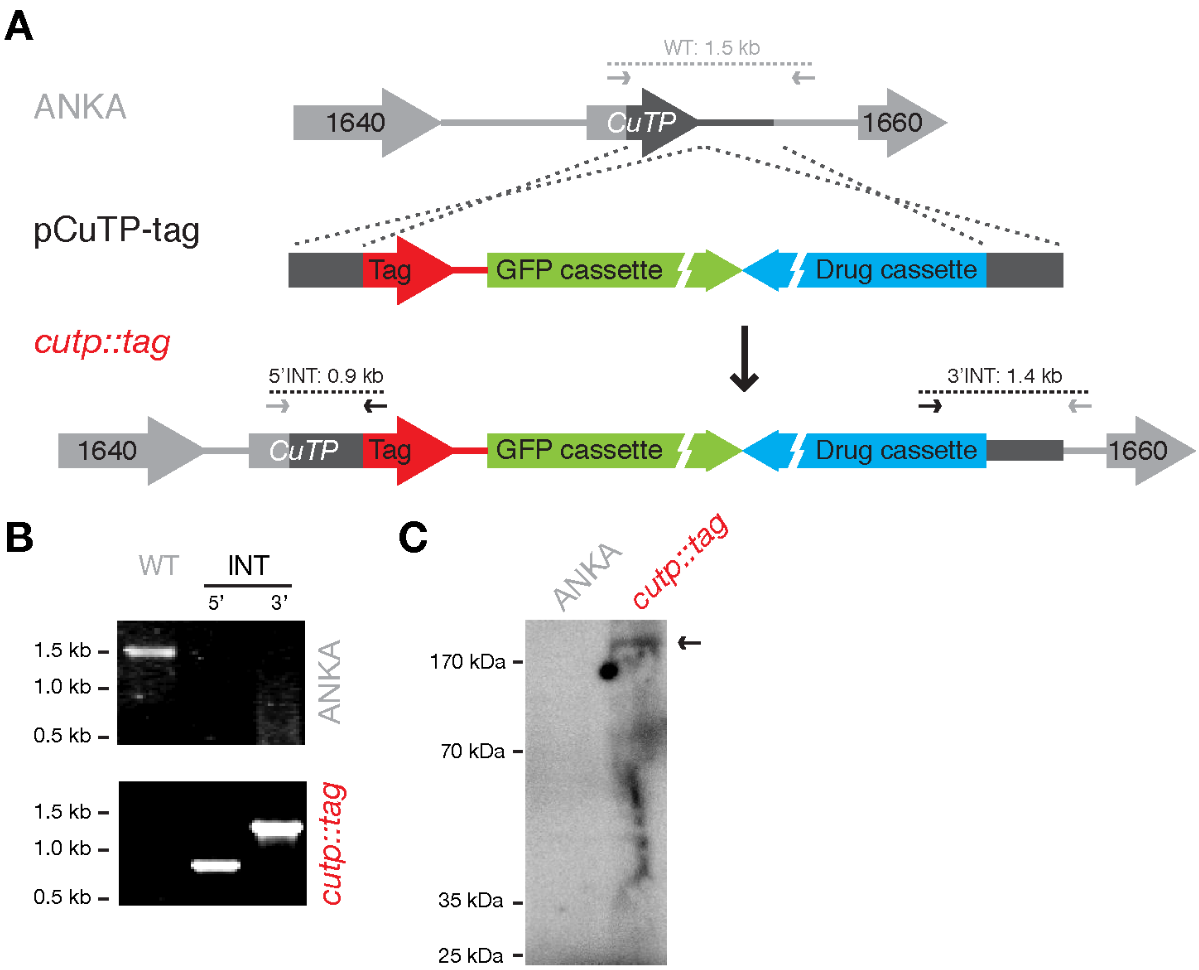


**Fig. S3.** Generation of *cutp::tag* parasites.

A. Replacement strategy to generate *cutp::tag* parasites. The carboxy-terminal and 3'UTR sequences of *CuTP* were cloned into the pBAT-SIL6 *P. berghei* transfection vector (pCuTP-tag). Upon an double cross-over homologous recombination event, *CuTP* is predicted to fuse in-frame to the mCherry-3xMyc tag and introduce the GFP and drug-selectable cassettes. Replacement (5'INT and 3'INT, black) and wild type (WT, gray)-specific test primer combinations and expected fragments are indicated.

B. Diagnostic PCR confirms successful integration of the CuTP-tag and absence of WT parasites after FACS of the isogenic *cutp::tag* parasite line.

C. Western blot analysis using anti-mCherry antibody shows absence of processed mCherry-3xMyc tag (33 kDa) and presence of a distinct band (black arrow) above 170 kDa indicative of the unprocessed CuTP::tag fusion protein (259 kDa).

**Kenthirapalan *et al*., Supplemental Figure S4**

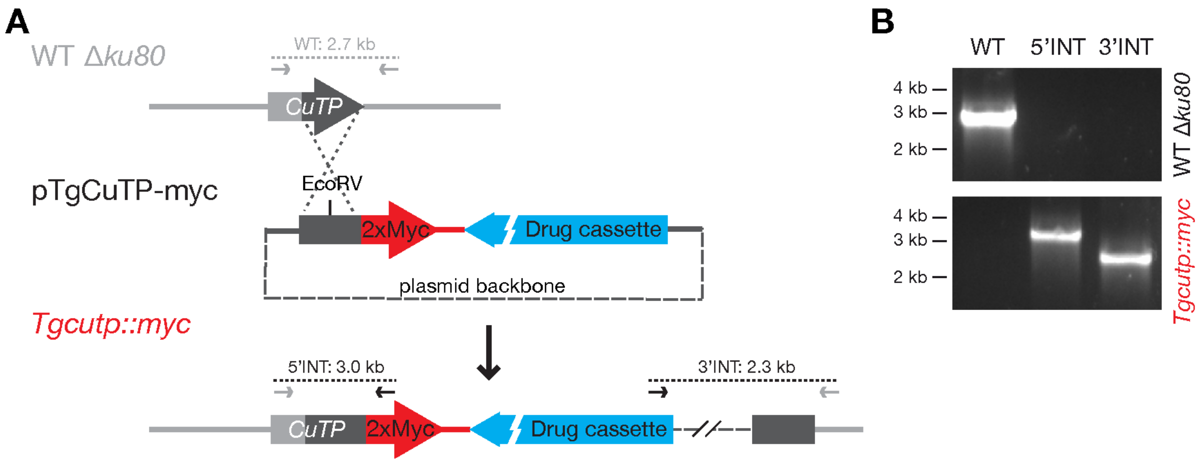


**Fig. S4.** Generation of *Tgcutp::myc* parasites.

A. Strategy used to generate endogenously tagged *Tgcutp::myc* parasites by single cross-over homologous recombination. The carboxy-terminal sequence of *TgCuTP* was amplified using gene-specific primers and cloned into the p5RT70mycGFP-DD/HX vector (pTgCuTP-tag). The resulting vector was linearized with EcoRV and used to transfect *T. gondii Δku80* parasites (WT). Insertion (5'INT and 3'INT, black) and wild type (WT, gray)-specific test primer combinations and expected fragments are indicated.

B. Diagnostic PCR confirms successful integration of pTgCuTP-myc and absence of WT parasites in the *Tgcutp::myc* parasite lines.

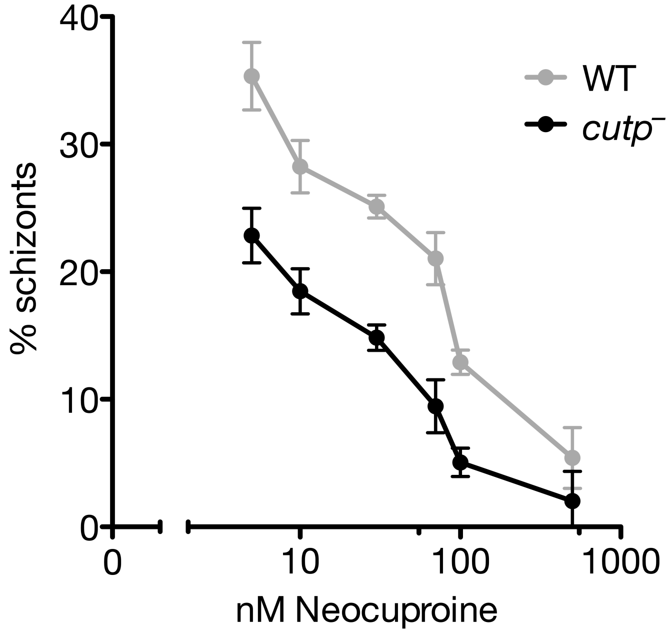


**Fig. S5.** The intracellular copper chelator neocuproine and loss of *CuTP* display additive effects on parasite maturation *in vitro*.

Addition of neocuproine over a range of concentrations inhibits schizont maturation in a dose-dependent manner to a similar extent in WT (gray) and *cutp*⁻ (black) parasites.

**Kenthirapalan *et al*., Supplemental Figure S6**

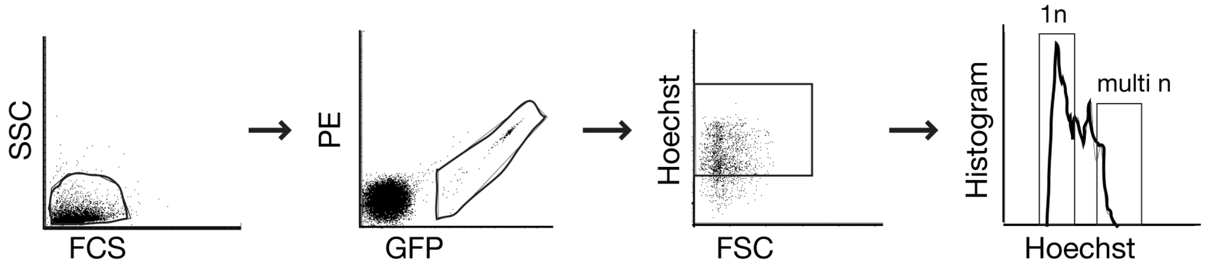


**Fig. S6.** Gating strategy for quantification of malaria parasite DNA.

Malaria parasite DNA content is a measure for the maturation of ring stage and trophozoite stage parasites (1n) to multinucleated schizont stages (up to 16n). The population distribution based on DNA content was determined using flow cytometry of Hoechst-stained 20 hour parasite cultures. First, small particles and debris and large cells were excluded using the side and forward scatter plot. Then GFP-positive cells were selected excluding auto-fluorescent cells and particles by gating for the $GFP^{hi}$ $PE^{lo}$ population. Finally, a histogram of Hoechst-positive cells was used to establish the size of the populations of cells with approximately 1n (ring, trophozoite, and sexual stages) and ≥8n (mature schizonts).

**Table S1.** Selected eukaryotic copper-transporting P-type ATPases.

| Clade | Species | Strain | Name | ID | Source |
|---|---|---|---|---|---|
| Kinetoplastida | ***Leishmania major*** | **strain Friedlin** | **LmCuTP** | **LmjF.33.2090** | EuPathDB |
| | ***Trypanosoma brucei*** | **Gambiense** | **TbCuTP** | **Tbg972.11.1330** | EuPathDB |
| | ***Trypanosoma cruzi*** | **CL Brener Esmeraldo-like** | **TcCuTP** | **Tc00.1047053511445.160** | EuPathDB |
| ALVEOLATA | *Plasmodium berghei* | ANKA | PbCuTP | PBANKA_041650 | EuPathDB |
| | *Plasmodium chabaudi* | chabaudi | PcCuTP | PCHAS_041740 | EuPathDB |
| | *Plasmodium yoelii* | yoelii 17XNL | PyCuTP | PY00066 | EuPathDB |
| | ***Plasmodium vivax*** | **Sal-1** | **PvCuTP** | **PVX_098690** | EuPathDB |
| | ***Plasmodium knowlesi*** | **strain H** | **PkCuTP** | **PKH_070210** | EuPathDB |
| | ***Plasmodium falciparum*** | **3D7** | **PfCuTP** | **PF3D7_0904900** | EuPathDB |
| | *Eimeria tenella* | str. Houghton | EtCuTP | ETH_00033450 | EuPathDB |
| | *Neospora caninum* | | NcCuTP | NCLIV_023240 | EuPathDB |
| | ***Toxoplasma gondii*** | **GT1** | **TgCuTP** | **TGGT1_020170** | EuPathDB |
| | *Cryptosporidium muris* | RN66 | CmCuTP | CMU_010800 | EuPathDB |
| | ***Cryptosporidium parvum*** | **Iowa II** | **CpCuTP** | **cgd3_3740** | EuPathDB |
| | *Perkinsus marinus* | ATCC 50983 | PmCuTP | XP_002778831.1 | GenBank |
| | *Tetrehymena thermophila* | SB210 | TtCuTP1 | EAR85041.1 | GenBank |
| | | | TtCuTP2 | EAR85040.1 | GenBank |
| PLANTAE | *Arabidopsis thaliana* | | AtHMA5 | NP_176533.1 | GenBank |
| | | | AtPAA1 | NP_974675 | GenBank |
| | | | AtPAA2 | NP_680181 | GenBank |
| | | | AtRAN1 | NP_199292.1 | GenBank |
| | *Chlamydomonas reinhardtii* | | CrCTP1 | XP_001699267.1 | GenBank |
| | | | CrCTP2 | XP_001702571 | GenBank |
| | | | CrCTP3 | XP_001697676 | GenBank |
| | | | CrCuTP | XP_001697676 | GenBank |
| FUNGI | ***Cryptococcus neoformans*** | **var. neoformans JEC21** | **CnCuTP** | **XP_568603.1** | GenBank |
| | *Saccharomyces cerevisiae* | S288c | ScCcc2p | NP_010556.1 | GenBank |
| ANIMALIA | ***Schistosoma mansoni*** | | **SmCuTP** | **XP_002575547.1** | GenBank |
| | *Caenorhabditis elegans* | | CeCUA-1 | NP_001255202.1 | GenBank |
| | *Anopheles gambiae* | str. PEST | AgATP7 | XP_552490.3 | GenBank |
| | *Drosophila melanogaster* | | DmATP7 | NP_572756.3 | GenBank |
| | *Mus musculus* | | MmAtp7a | NP_001103227.1 | GenBank |
| | | | MmAtp7b | NP_031537.2 | GenBank |
| | *Homo sapiens* | | HsATP7A | NP_000043 | GenBank |
| | | | HsATP7B | NP_000044.2 | GenBank |
| Outgroup | *Escherichia coli* | K12 | EcCopA | Q59385 | GenBank |

**Table S2.** Primer sequences.

| Primer Name | Primer Sequence (restriction sites are underlined) |
|---|---|
| CuTP-F1 | ATTAATATTATAAGCCCAATAAAATGTACG |
| CuTP-F2-SacII | AAACCGCGGGAACTATTTCTCCGTATAACAGG |
| CuTP-F3 | TTGGGAATACCAAAAAGCAACG |
| CuTP-F4-SacII | AAACCGCGGTTTTGCCTTGAAAACTGCTG |
| CuTP-F5-AvrII | AATCCTAGGCGCTTTGCCAAATTTCATTTTATC |
| CuTP-R1-EcoRI | AAAGAATTCTTAATTAATATAATATATGTTCATACACTAATATGACCC |
| CuTP-R2 | TCGAAGCAGATTTATTTGTAAAGG |
| CuTP-R3-PshAI | TAAGACATATGTCTACATTTTTCAGAATCCTATAAATGTGTAA |
| CuTP-R4-KpnI | ATAGGTACCACTATGTATGTGCGCATTATTTTC |
| CuTP-R5 | TCAATGAAATCCAACTACTCATTCC |
| SIL6F[a] | GACAGCGCATATGATGGATG |
| SIL6R[a] | TACGAATACGCAATTTCTCAAAC |
| 5'HSP70rev[a] | CAATTTGTTGTACATAAAATAGGCAG |
| 5'DHFRrev[a] | ATGAAATACCGCTCCATTTTTCC |
| mCherryRev[b] | CCCTCCATGTGAACCTTGAAG |
| TgCuTP-F1 | CTCAGATCTTGTCCAAACAAAGC |
| TgCuTP-F2-ApaI | ATAGGGCCCGCTGAAATCTCTCTGCCAGT |
| TgCuTP-R1-2xmyc-PacI[c] | ATATTAATTA**AAGATCCTCTTCAGAAATTAATTTTTGTTC**AGCAGC**TAAATCTTCTTCAGAAATTAATTTTTGTTC**TCCAGCGTCTGCGTGTTCTCG |
| TgCuTP-R2 | CATGAAACCCAAATACGTTAAGC |
| 3'TgHXGPRTfor | CAGTGCGCATAAAAATGTGG |
| T7 | TAATACGACTCACTATAGGG |

[a] Reference: (Kenthirapalan *et al.*, 2012)
[b] Reference: (Haussig *et al.*, 2011)
[c] Sequences encoding the two c-myc epitopes are in bold

## Supplemental Experimental Procedures

### Cloning of plasmids pCuTP-KO and pCuTP-tag

First, a 486 bp fragment of the 3'UTR was amplified from gDNA using the primer combination CuTP-F5-AvrII and CuTP-R4-KpnI (see Table S2 for all primer sequences) and cloned into the pBAT-SIL6 vector (Kooij *et al.*, 2012) using AvrII and KpnI to generate the intermediate construct pCuTP-IM. For the generation of the *CuTP* disruption construct (pCuTP-KO), a 561 bp fragment of the 5'UTR was amplified from gDNA using the primer combination CuTP-F2-SacII and CuTP-R1-EcoRI. This fragment was subcloned using EcoRI and SacII into an intermediate pBAT-derived vector. Subsequently, the *CuTP* targeting fragment was released by cleavage with HpaI and SacII and cloned into pCuTP-IM using PvuII and SacII.

For the tagging construct, termed pCuTP-tag, a 498 bp fragment of the carboxy-terminal coding region of *CuTP* was amplified from gDNA using the gene-specific primers CuTP-F4-SacII and CuTP-R3-PshAI and fused in frame to the mCherry-3xMyc tag of pCuTP-IM using SacII and HpaI.

### Diagnostic genotyping PCR

To demonstrate successful integration and isogenic background of the selected parasite lines, the following specific primer combinations were used (see Table S2 for primer sequences): CuTP-F1 and 5'HSP70rev (5' integration of pCuTP-KO, 981 bp), CuTP-F3 and mCherryRev (5' integration of pCuTP-tag, 908 bp), 5'DHFRrev and CuTP-R5 (3' integration of both vectors, 1,456 bp), CuTP-F1 and CuTP-R2 (5' WT for the control of *cutp*$^{-}$ only, 948 bp), and CuTP-F3 and CuTP-R5 (3' WT, 1,440 bp).

### Western blot analysis

Whole protein extracts of mixed blood stage WT and *cutp::tag* parasites were isolated and separated on a 8 % SDS-polyacrylamide gel. The separated proteins were blotted on a PVDF membrane, incubated with rat monoclonal anti-mCherry antibodies (1:1000; ChromoTek) and detected with horseradish peroxidase coupled goat anti-rat antibodies (1:5000; Jackson ImmunoResearch).

### Cloning of the pTgCuTP-myc plasmid

A 2.3 kb fragment of the carboxy-terminal region of *TgCuTP* was amplified from genomic DNA using the primer combination TgCuTP-F2-ApaI and TgCuTP-R1-2xmyc-PacI. The resulting fragment was cloned into the p5RT70mycGFP-DD/HX vector (Herm-Götz *et al.*, 2007) using restriction digestion with ApaI and PacI, thus replacing the 5'*TUB-Myc-GFP-DD* sequence. Prior to transfection, the resulting pTgCuTP-myc plasmid was linearized overnight by restriction digestion with EcoRV and purified via ethanol precipitation. Integration of pTgCuTP-myc is thus predicted to result in the carboxy-terminal tagging of the endogenous *TgCuTP* fused in-frame to a double c-Myc epitope tag followed by the 3' untranslated region of *TgSAG1* along with insertion of the *HXGPRT* drug-selectable cassette.

### Diagnostic genotyping PCR of *Tgcutp::myc*

To demonstrate successful integration of the selected parasite line, the following specific primer combinations were used (see Table S2 for primer sequences):

TgCuTP-F1 and 3'TgHXGPRTfor (5' integration, 3.0 kb), T7 and TgCuTP-R2 (3' integration, 2.3 kb), TgCuTP-F1 and TgCuTP-R2 (WT, 2.7 kb).

### *In vitro* drug inhibition assay

To determine the distribution of asexual blood stages *in vitro*, mice were infected with purified schizonts and bled after 2 h. The resulting synchronized ring stages were cultured in the absence or presence of the intracellular copper chelator neocuproine (Sigma-Aldrich) for 20 h, as indicated. Thereafter, DNA was stained with Hoechst 33342 (Invitrogen) and the intensity of the nuclear stain analyzed using a MACSQuant Analyzer (Milteny Biotec; Fig. S6). The red blood cell population was gated to exclude small debris and large cells using the forward scatter versus side scatter plot. This subpopulation was further gated for GFP-positive events. The Hoechst-positive cells of the GFP-positive subpopulation were divided into two subpopulations, according to their DNA content, *i.e.* 1n and multi n (≥8n).

## Supplemental References


Argüello, J.M. (2003) Identification of ion-selectivity determinants in heavy-metal transport P1B-type ATPases. *J Membr Biol* **195**: 93-108.

Felsenstein, J. (1996) Inferring phylogenies from protein sequences by parsimony, distance, and likelihood methods. *Meth Enzymol* **266**: 418-427.

Haussig, J.M., Matuschewski, K., and Kooij, T.W.A. (2011) Inactivation of a *Plasmodium* apicoplast protein attenuates formation of liver merozoites. *Mol Microbiol* **81**: 1511-1525.

Herm-Götz, A., Agop-Nersesian, C., Münter, S., Grimley, J.S., Wandless, T.J., Frischknecht, F., and Meissner, M. (2007) Rapid control of protein level in the apicomplexan *Toxoplasma gondii*. *Nat Methods* **4**: 1003-1005.

Kenthirapalan, S., Waters, A.P., Matuschewski, K., and Kooij, T.W.A. (2012) Flow cytometry-assisted rapid isolation of recombinant *Plasmodium berghei* parasites exemplified by functional analysis of aquaglyceroporin. *Int J Parasitol* **42**: 1185-1192.

Kooij, T.W.A., Rauch, M.M., and Matuschewski, K. (2012) Expansion of experimental genetics approaches for *Plasmodium berghei* with versatile transfection vectors. *Mol Biochem Parasitol* **185**: 19-26.
